# Eating away at food allergy

**DOI:** 10.1111/pai.70251

**Published:** 2025-12-01

**Authors:** Yue Jennifer Du, Jessica C. Guo, Julia E. M. Upton

**Affiliations:** ^1^ Internal Medicine, DeGroote School of Medicine McMaster University Hamilton Ontario Canada; ^2^ Temerty School of Medicine University of Toronto Toronto Ontario Canada; ^3^ Division of Immunology & Allergy, Department of Paediatrics, The Hospital for Sick Children University of Toronto Toronto Ontario Canada

**Keywords:** biologics, food allergy, immunotherapy, peanut allergy, prevention

## Abstract

Food allergy (FA) is a significant public health concern, with its prevalence rising globally and greatly affecting the lives of patients and their families. The increasing burden on healthcare systems and the impact on quality of life underscore the need for better understanding and management strategies. The dual‐allergen hypothesis suggests that early skin exposure to allergens increases sensitization risk, while early oral exposure and sustained ingestion of foods promote tolerance While diet is not the only factor in FA development, eating allergenic foods early and often can profoundly prevent FA despite other risk factors such as eczema. Treatment approaches vary by a number of factors including patient preference. Avoidance remains an option, but tailored avoidance, such as allowing denatured food products with milk and egg is now commonplace. Food immunotherapy approaches via multiple routes and doses are becoming more available. Immunotherapy can result in marked reductions in food reactivity which may be sustained for weeks or months or even longer off treatment for some patients. Biologics are also being more widely offered to increase the amount of food that can be safely ingested to facilitate immunotherapy. With the current approaches, treatment at a time of low IgE formation, often associated with younger age, may be the most effective for remission, but older ages may benefit from the increase in the threshold of reactivity that food‐based treatments can provide.

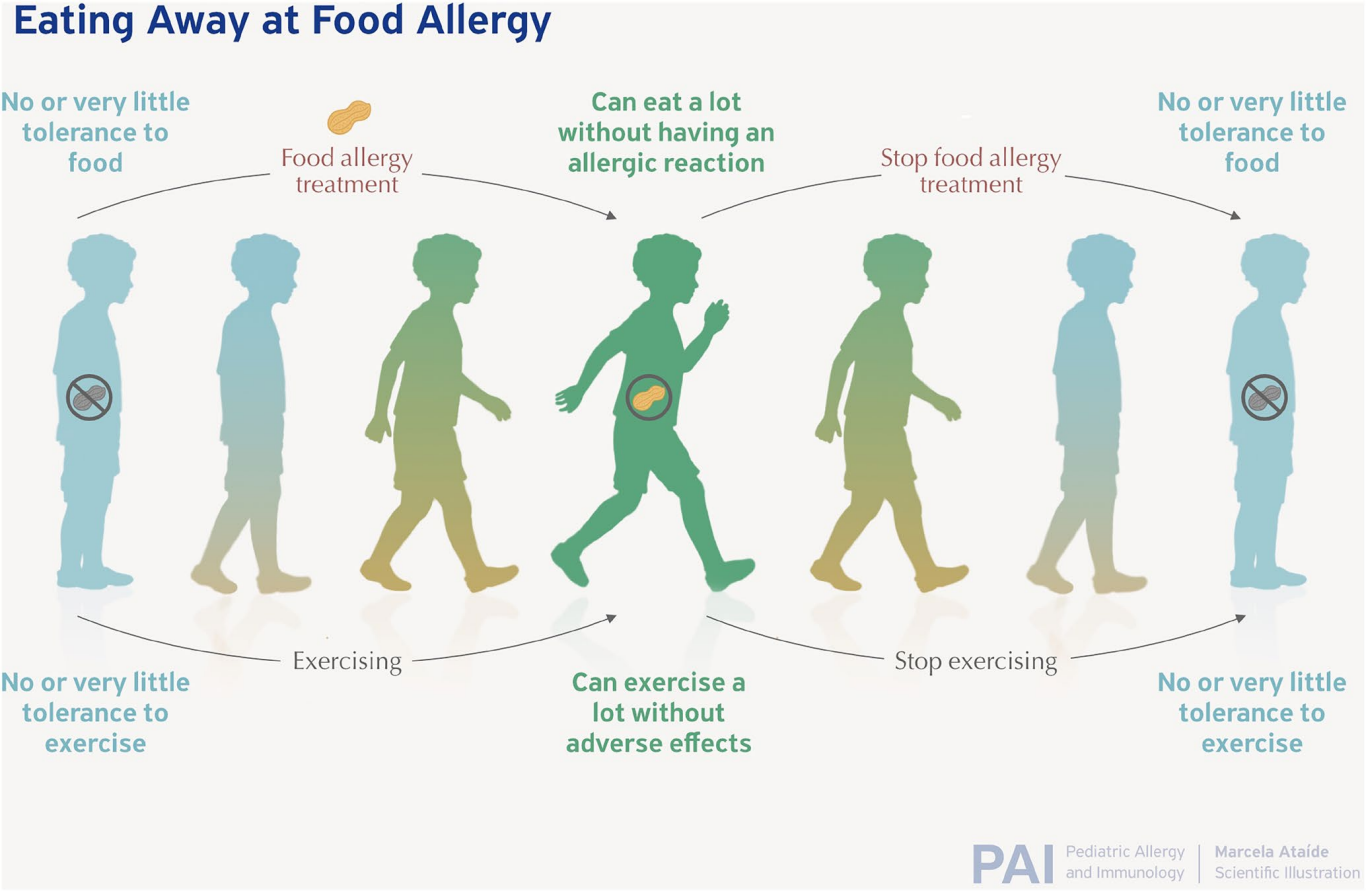

## Graphical abstract

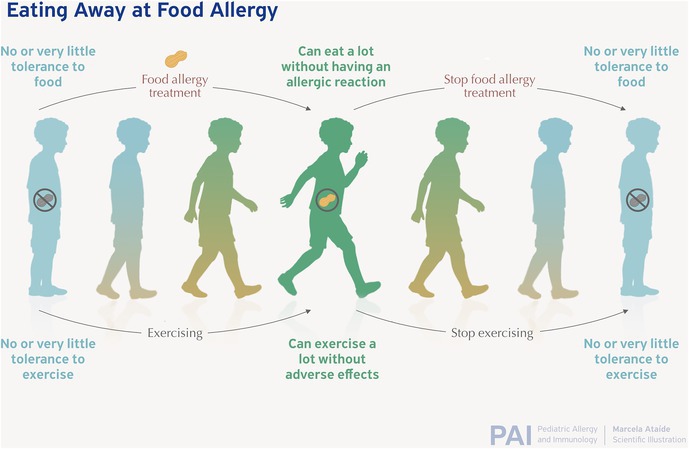

**Key message**

The gut is designed to tolerate foreign proteins. The prevention of FA has been best demonstrated by an approach to ingest foods, including the commonly allergenic foods, early in infancy. Increasingly it is being recognized that it is important to keep these foods in the diet. Intermittent exposure to allergenic foods may be worse than avoiding the food. Therefore, we need to promote a message to also routinely eat the introduced foods. The treatment of FA, much like its prevention, can also be to expose the immune system to the foods early and often. This exposure reduces reactivity to the food with the most data for oral immunotherapy and can also be achieved by other routes of exposure (under the tongue or by consistent exposure through the skin). The future of food‐based immunotherapy treatment includes further refining these treatments to balance safety and efficacy and the selected use of medications including biologics.

## 1 INTRODUCTION AND BACKGROUND

Food allergy (FA) is increasingly common worldwide and is the leading cause of anaphylaxis in children.^1^ Globally, the prevalence of FA is around 6%, ranging from 2% to 10%.^2^, ^3^ FA utilizes a significant proportion of healthcare dollars in the developing world and significantly affects quality of life.^4^

In a North American, Canadian context, about 7.5% of the population is living with one or more FA^5^ and more than 50% of Canadians are directly or indirectly impacted.^6^ With a population of over 40 million people in 2023, the impact of FA on Canadians is significant. Among Canadian children, the most commonly documented allergens include peanut (0.8% of children), tree nut (0.6%), cow's milk (0.4%), and egg (0.3%).^7^

FA makes up a large component of Canadian health spending.^8^ The estimated cost of FA annually totaled 41.9 billion dollars, including $2.8 billion in healthcare, $4.9 billion in out‐of‐pocket costs, and 34.4 billion in indirect costs.^8^

In the last decade, there has been an increase in our understanding of the pathophysiology and management of allergy, leading to many developments in both its treatment and prevention. Specifically, the areas of prevention of FA by early introduction, food ladders, oral immunotherapy (OIT) and the use of biologic medications have been focuses of research. In this review, we focus on the key role of exposing food allergens to the gastrointestinal tract, both as prevention of and as treatment of FA. Important gaps remain in the pathophysiology of food allergy development as it relates to food exposure and optimal food‐based protocols for prevention and treatment.

### 1.1 Pathophysiology of FA

Many theories have shaped our understanding in the development of allergic diseases, The Hygiene Hypothesis suggests that reduced microbial exposure and diversity early in life due to increased cleanliness, smaller family sizes, and urban settings impairs the development of immunoregulatory pathways and increases the risk of allergies.^9^ The Old Friends hypothesis builds on the hygiene hypothesis and proposes that microbes have co‐evolved with humans and early exposure may help prevent immune dysregulation.^9^

The Epithelial Barrier Hypothesis is a broader framework that links modern environmental exposures (pollutants, detergents, and dietary changes), to epithelial barrier damage in the skin, gut, and airways. This damage increases permeability, enabling allergens and microbes to enter and activate type 2 immune responses, chronic inflammation, and dysbiosis.^10^

**FIGURE 1:**
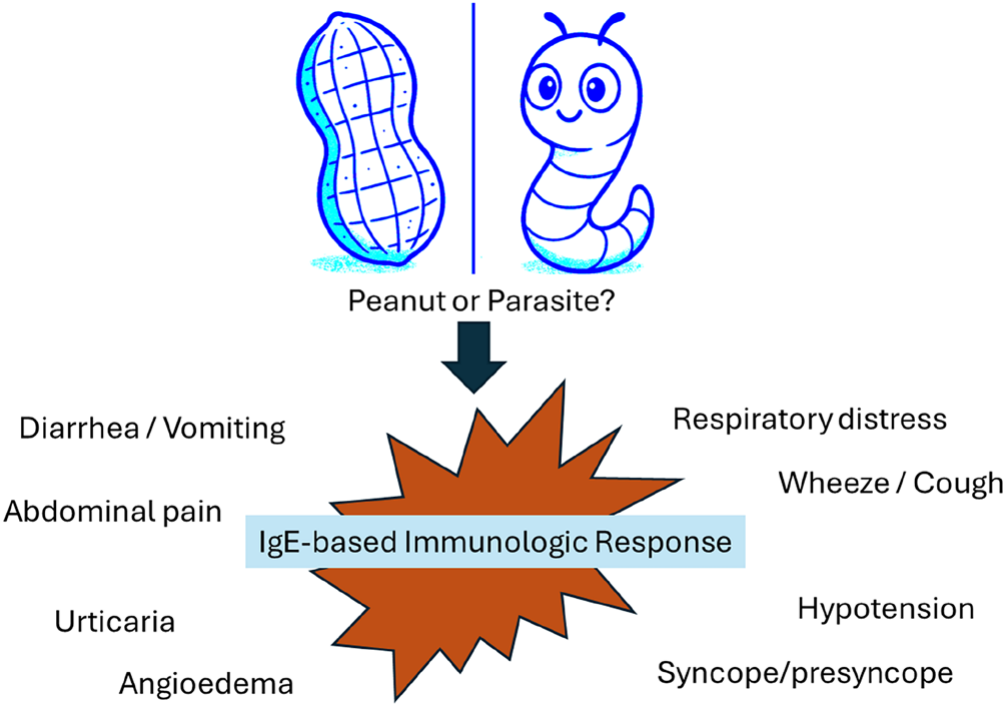
IgE‐mediated allergy occurs when a parasitic immune response is inappropriately activated against foods.

Historically, delaying the introduction of allergenic foods into the diets of infants and young children was thought to prevent FA.^11^ One prior theory is that food allergies arise from the gut when there is a failure of oral tolerance due to impaired intestinal permeability, as was recently reviewed.^12^ The theory of gut permeability as a route of allergy has not held. It has been demonstrated that children with peanut sensitization (positive IgE antibody allergy tests) or clinical allergy (reaction to the food when ingested) did not have higher rates of impaired intestinal permeability compared to control populations nor was permeability altered by peanut introduction.^12^ It is now recognized that early oral exposure can establish and maintain tolerance to foods. Allergen exposure in the absence of oral ingestion has emerged as the main route of sensitization. This theory has been referred to as the dual allergen hypothesis.^13^ This theory postulates that food allergies develop due to a disrupted skin barrier which allows the immune system to be exposed to the food and interpret it as an invader. The immune system then develops IgE antibodies towards the food. When subsequently exposed orally, the immune system leads the attack to expel the food with symptoms of vasodilation vomiting, and bronchoconstriction. This response mimics the immune system's defense against parasites (Figure 1). The dual allergen hypothesis may not explain all cases of FA development. One route of sensitization which may be important as demonstrated in mouse models is airway exposure to allergens prior to oral exposure.^14^ Studies continue investigating the role the gut microbiome plays in allergy development.^12^, ^13^

### 1.2 Early exposure to foods is protective against allergy

The landmark randomized controlled trial Learning Early About Peanut (LEAP) study found that introducing peanuts to infants age 4–11 months at high risk for developing peanut allergy (severe eczema and/or egg allergic) yielded more than an 80% decrease in peanut allergy at age 5 years compared with children who avoided peanuts until 5 years of age.^15^ This study showed that early ingestion promotes immune tolerance.

The EAT study further evaluated if the early introduction of common allergic foods (peanut, cow's milk, cooked egg, sesame, whitefish, and wheat) led to a decrease in the incidence of food allergies compared to a group with standard food introduction. A nonsignificant 20% lower relative risk for those who had early exposure to the outlined allergens was found in the intention‐to‐treat analysis; the per‐protocol analysis found a significant 67% lower relative risk of FA. Notably, only peanut and egg showed significant reduction in FA, although there may not have been a high enough rate of allergy to the other foods to draw conclusions.^16^ Importantly, early food introduction did not interfere with breast‐feeding.^17^

The protective effect of early oral exposure aligns with our understanding that the immune system is designed to distinguish between harmful pathogens and harmless foreign antigens such as food proteins. The immune system has evolved to be able to encounter foreign antigens via the GI tract; oral exposure is a normal and anticipated route of exposure to foreign proteins. The gut‐associated lymphoid tissue (GALT) is adapted to promote immune tolerance by inducing regulatory responses to dietary antigens and commensal microbes.^18^ The ability to tolerate non‐self proteins introduced via the gut is essential for survival because without this tolerance, we would not be able to eat any food without adverse effects. Early introduction of allergenic foods helps the immune system learn that these proteins are harmless, essentially teaching it that “food is food.”

### 1.3 Early and sustained exposure to foods is required to maintain tolerance

There is mounting evidence that not only early introduction is important, but continued exposure is also key to promoting immune tolerance to foods. In a follow‐up study of LEAP, 4 years of regular consumption of peanut induced stable non‐reactivity to peanut as assessed after 12 months of intermittent ingestion/avoidance.^19^ However, the Cow's Milk Early Exposure Trial (COMEET) study^20^, ^21^ enrolled pregnant people and tracked feeding methods (formula or exclusive breast feeding (EBM)) and assessed cow's milk allergy in the infants. None of the children (0/919) fed cow's milk formula routinely during the first 2 months of life developed cow's milk allergy at 12 months. Cow's milk allergy occurred only in the EBM group (17/1073). Importantly, 13/17 of the children who developed allergy had been briefly supplemented with cow's milk formula in the first days of life, suggesting that intermittent exposure at early ages may be sensitizing. Another study also demonstrated that inconsistent and infrequent exposures to small amounts of cow's milk may lead to an increased risk of cow's milk allergy development. Sakihara et al.^22^ demonstrated that cow's milk allergy is associated with discontinuation of cow's milk exposure within the first month of life. Prevention of cow's milk allergy presents a unique challenge because while the evidence for a reduction in cow's milk allergy with early and often cow's milk formula exposure is mounting, implementation becomes complicated with EBM guidance and the international code of marketing of infant formula and other products used as breast‐milk substitutes.^23^ Canadian messaging highlights the risk of intermittent exposure and advises that if cow's milk formula is used to consider continuing some ongoing exposure.^24^, ^25^, ^26^

The lessons learned from the prevention studies are that it is protective against FA to expose the immune system to food with both early (after 4 months of age, when developmentally ready) and ongoing oral exposure.^21^, ^24^, ^25^, ^27^ In Canada, the Eat Early, Eat Often patient education initiative aims to disseminate this message.^28^

Further evidence for the importance of early introduction and ongoing ingestion is demonstrated with biomarkers from the LEAP study.^15^ Peanut‐specific IgE rose overtime in children eating or avoiding peanut; however there were only very high peanut IgE levels in those avoiding. Furthermore, IgG4 levels, often considered a blocking allergy against peanut allergy,^29^ were minimal or absent at 4–11 months in both groups and rose at a higher rate in the early peanut exposure group than in the avoidance group. This rise in food specific IgG4 is also seen in the treatment of FA by immunotherapy.^30^, ^31^

## 2 TREATMENT

Ideally, the prevalence of food allergies will decrease with widespread prevention measures. However, there will still be some allergic children. Traditionally, food avoidance was the only recommendation to manage food allergies. Elimination diets pose many adverse effects to patients as they could lead to nutritional deficits, perpetuate the fear around food, and impinge on quality of life (QOL). Avoidance diets are also not completely effective as evidenced by accidental exposure to peanut in peanut‐allergic children of over 10%.^32^ In the recent decade, there have been many advances in the therapies for the treatment of food allergies. Two of the major advances in management focusing on food‐based therapies are exposure to the food and the adjunctive use of biologic medications.^33^, ^34^

### 2.1 Ingestion of tolerated forms/amounts

#### 2.1.1 Baked milk and Egg

Milk and egg allergies are still some of the most common in the pediatric population. Prognosis is good with about 80% of children outgrowing milk allergy and 50% outgrowing their egg allergy by age 6 years.^35^ Both foods are changed by heat causing denaturing of some of the proteins.^36^ It is estimated that more than 70% of cow's milk or egg allergic children can tolerate the food in a bakery product, meaning for example a well‐cooked wheat flour‐based muffin. Allergists offer “baked challenges” to assess if a child can ingest this well‐cooked form and therefore allow some dietary restrictions to be lifted.^36^

**FIGURE 2:**
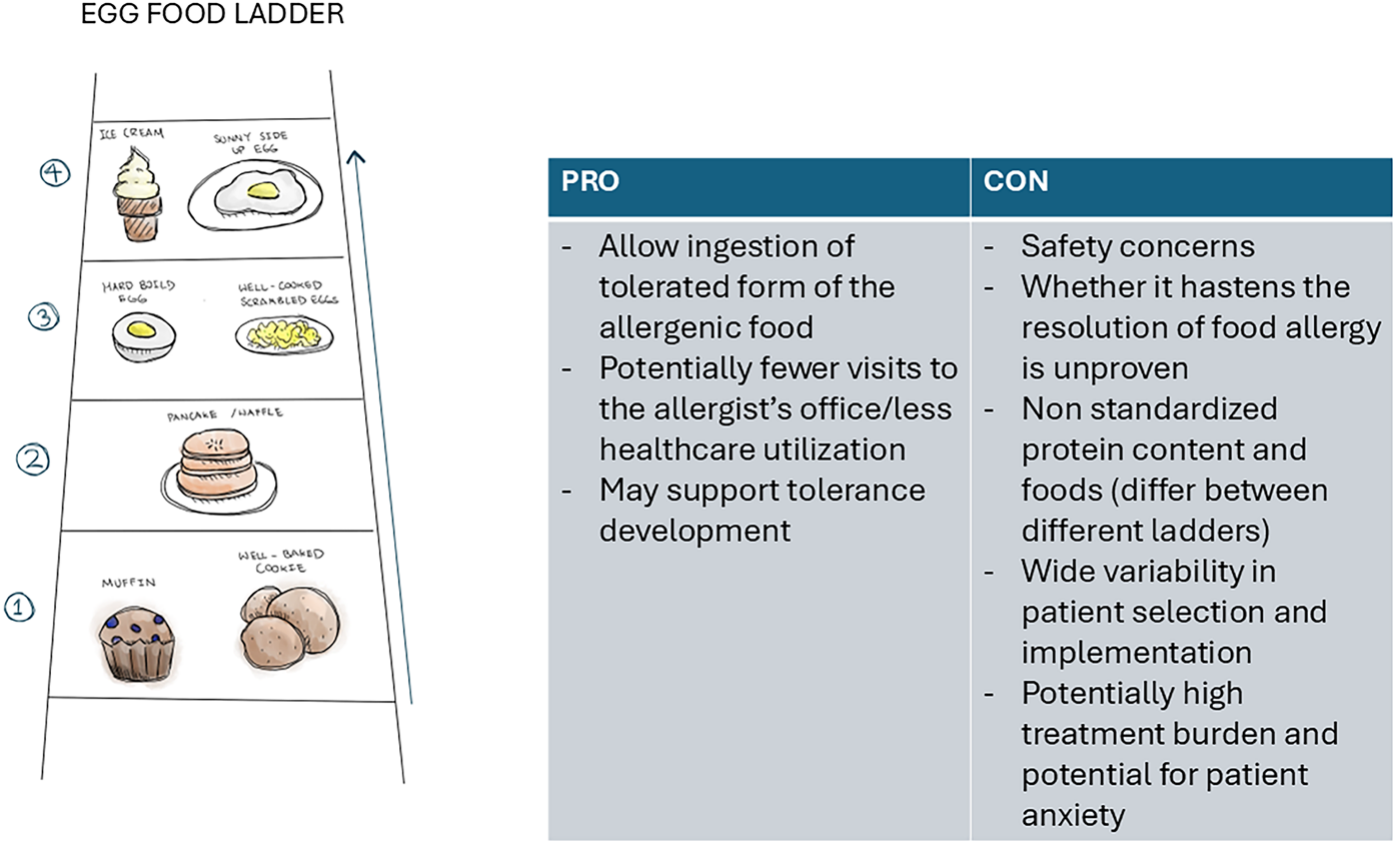
An example of an egg food ladder and considerations for its use.

A further application of this concept of heating changing the allergenicity is found in milk and egg ladders. The “ladder” involves the stepwise administration of more heated to less heated forms of the allergic protein and was originally developed for home reintroduction of cow's milk in non‐IgE mediated FA.^36^ In Canada, food ladders are widely used to advance diets in children with IgE‐mediated allergy.^37^ There is still a risk of anaphylaxis with this approach. A small number of participants in one survey noted use of epinephrine (2/53, 3.8%) during the process of the food ladder. Minor reactions such as skin rash were reported in the majority of participants (10/14) while no patients reported severe reactions.^38^ Ireland also widely reports the safe use of cow's milk ladders in very young children with cow's milk allergy.^39^, ^40^ Importantly, a meta‐analysis involving 29 studies found that there was very low certainty evidence supporting their efficacy and safety^41^ and another study found there was significant practice variability.^42^ If the ladder is initiated with no knowledge of a child's tolerance to baked forms of milk or egg, some will be allergic to the baked form and therefore have a risk of severe reactions.^36^ Ways to improve safety may be to limit the introduction of baked foods to medically supervised settings or to restrict home dietary advancement via the ladder approaches to compliant families with preschool or younger children who have not had a severe reaction or risk factors for severe reactions (including asthma).^43^ There have also been calls to standardize the foods.^44^ (Figure 2).

#### 2.1.2 Threshold Dose Testing

Establishing the threshold amount of protein which may lead to reaction is key to understanding treatment effects and also increasing patients' confidence with eating and exposures.^45^, ^46^ Threshold dose varies by definition but is essentially the amount of food protein that will cause allergic symptoms in an allergic individual. Several biomarkers have some correlation with threshold dose. Particularly, an inverse relationship has been noted with characteristics of allergen specific serum IgE and basophil activation tests while a positive relationship has been observed with pathogenic effector TH2 cell levels.^46^, ^47^ These markers are imperfect, and the gold standard is to perform an oral challenge to determine an individual's threshold dose. Although the threshold dose can vary in the same individual depending on other factors (such as exercise and illness), one study found that most untreated patients had a similar threshold dose on repeated challenge.^48^

#### 2.1.3 Potency of allergenic foods

**FIGURE 3:**
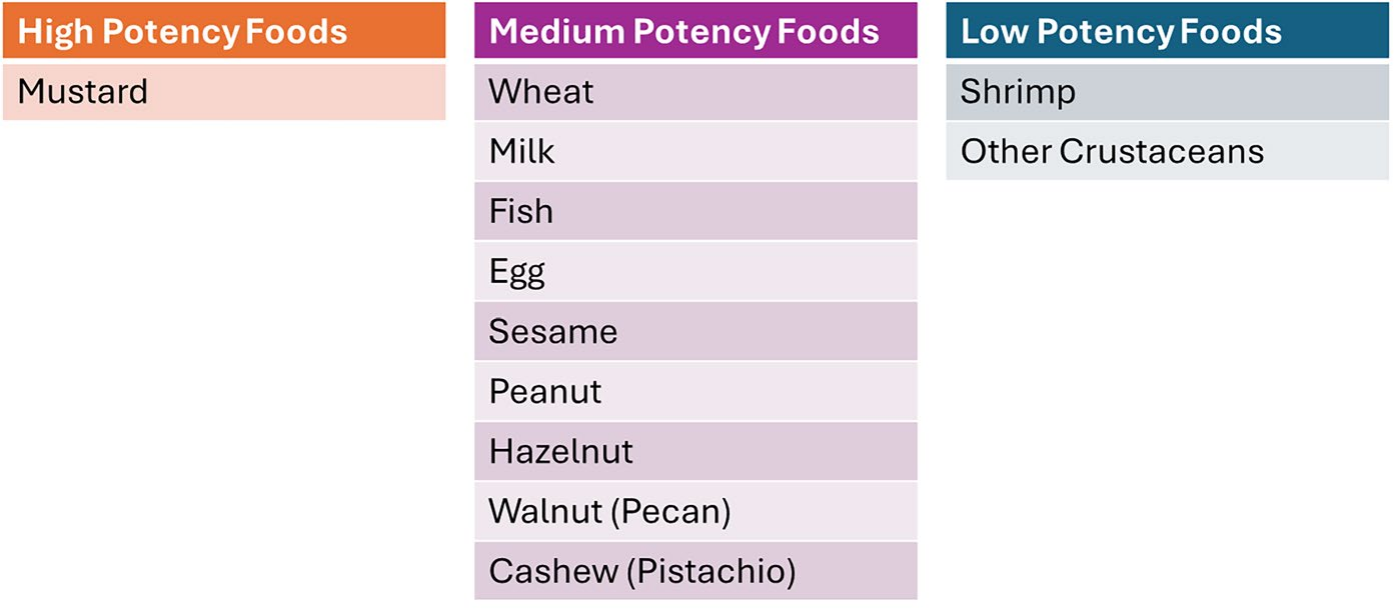
Different potencies (referring to the amount of protein per volume of food that causes an allergic reaction in allergic individuals) of different foods. High potency means at a population level a small amount can trigger an allergic reaction.

The potency of different foods, describing the amount of protein which elicits an allergic reaction in a given proportion of the population, differs by food (Figure 3).^49^ In addition to labelling foods, use of thresholds has been utilized in considering and individualizing therapies such as immunotherapy and biologics.^46^ They may also allow patients and families who previously had lots of anxiety around public eating to have the confidence to incorporate new food experiences into their lives.^46^

### 2.2 Immunotherapy to food

**FIGURE 4:**
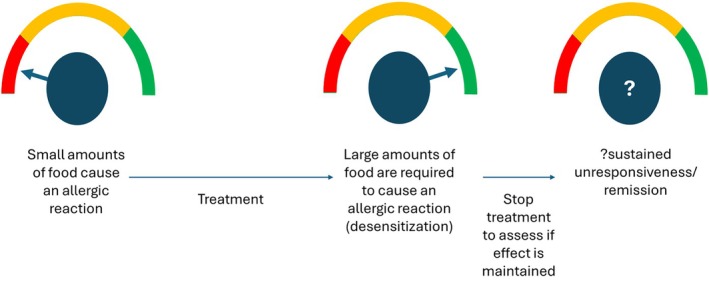
Threshold dose is typically established to be the amount of food protein that will cause allergic symptoms in an individual. It is a spectrum among individuals and can change in a specific individual depending on cofactors present and be increased by treatments. The threshold can be assessed after stopping treatment to see if the treatment effect has been maintained.

Immunotherapy has been used for close to a century to treat allergies. The main principle is that repeated and consistent administration of gradually increasing amounts of an allergen will change the response of the immune system and allow it to be exposed to a higher amount without a reaction. This increase in the threshold of reactivity is called desensitization. This method was initially used for plant pollen and insect venom allergies to good effect. Food immunotherapy is now rapidly increasing utilization in the treatment of FA.^50^ Treatment effect can be assessed after a period of treatment to discover if there has been a durable effect called remission or sustained unresponsiveness (Figure 4).

#### 2.2.1 Oral Immunotherapy

OIT is the ingestion of food at, ideally, sub‐threshold amounts such that the immune system response can be changed without triggering an allergic reaction. In practice, the threshold can change due to co‐factors, as described above, as well as external factors such as medications. In a meta‐analysis looking at the efficacy and safety profile of OIT, many studies pointed to OIT being effective in increasing the threshold of patients with food allergies. The main adverse effects included an increase in local and systemic adverse events (including anaphylaxis) to the OIT dose and a risk of eosinophilic esophagitis (EoE).^33^ Although EoE is a rare complication, it can lead to long‐term complications with persistent symptoms and chronic esophageal inflammation leading to fibrosis. EoE can present months to years after OIT initiation, and while most cases resolve after discontinuation of OIT, some patients may require treatment for persistent EoE.^51^, ^52^

In Canada, the risks and benefits were reviewed including a lens of ethical, evidence‐based and patient‐oriented factors and the recommendation in 2020 was that OIT should be carefully offered to those who want it, with no exclusions due to prior reaction severity and to any food and any age.^53^

Several factors improve the odds of success with immunotherapy for FA and potentially reduce adverse effects Age is one such factor. The IMPACT study found that in children with peanut allergies, initiation of OIT before the age of 4 years was associated with improved rates of peanut tolerance and remission of allergy, with a suggestion that the most remission occurred if OIT was initiated before age 2 years.^54^ This beneficial effect may be more complex than age because the youngest children also had the lowest peanut specific‐IgE tests. Even children who did not achieve full remission demonstrated a higher reactivity threshold for peanut protein. There was a large percentage of patients who experienced side effects in the OIT and placebo groups (98% vs. 80%, respectively). Most of the reactions were mild–moderate although 21/96 children who had moderate reactions did receive epinephrine administration. Ninety‐six participants with persistent gastrointestinal symptoms after OIT underwent endoscopies and 3% (3/96) of participants were found to have EoE often after discontinuation of therapy. Whether this EoE was due to the OIT or an unmasking of a previously quiescent condition is unclear.^55^ Overall, this trial exemplifies “re‐learning” of the immune system during an opportunistic window of time in the development of the immune system. There is currently a push in Canada to try to treat the youngest children soon after diagnosis.^56^ When sensitization and reactivity have already occurred, reintroducing the allergen in a gradual, controlled way may help to retrain the immune system. Even if complete resolution of allergy is not achieved, early and sustained exposure shifts the immune response towards increasing tolerance.

It is important to note that OIT has worked in older age groups as well. Desensitization rates are similar to children and adolescents for many foods except cow's milk.^57^, ^58^ Additionally, when controlled for age, it is the sIgE that is important for treatment outcome.^57^, ^58^

There are many different protocols for OIT with local and international variations. The optimal dose remains to be determined.^59^ A pivotal study by Vickery et al. suggested that both 300 mg (approximately 1 peanut) and 3000 mg gave similar results in terms of desensitization outcomes, although it was not powered to directly compare.^60^ It is important to note that these children were not only young (up to age 4 years old), but were also recently diagnosed. In Japan there is great interest in low and slow dose OIT for the treatment of peanut allergy.^61^ As an example, Nagakura et al.'s team studied children with a history of anaphylaxis and found that increasing doses of peanut protein up to 133 mg/day over the course of 1 year (about half a peanut) demonstrated sustained unresponsiveness, after 2 weeks of peanut avoidance, in an oral challenge to 795 mg of peanut protein.^62^ No participants in the placebo group were found to have sustained unresponsiveness. This suggests that low dose OIT over a longer period of time may be a reasonable therapeutic option for high‐risk peanut allergy patients to implement a higher threshold of reaction and prevent severe anaphylactic reactions to accidental exposures.^62^ Recent work from Canada showed that only 30 mg of peanut protein increases the threshold of allergic reaction compared to strict avoidance in children with a median age of 10 years.^63^ Low doses of food may be one way to teach the immune system that food is food while minimizing adverse effects and reducing medical visits.

**FIGURE 5:**
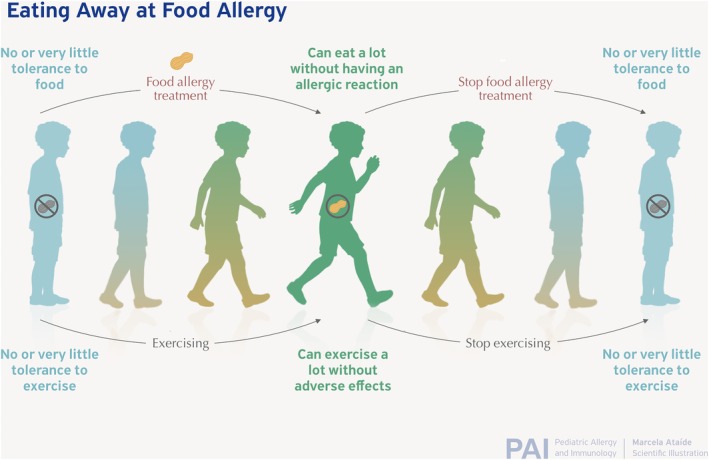
Fitness/endurance as an analogy to food desensitization. Both involve exposing the body to an activity initially poorly tolerated to gain capacity: In food treatments this is the capacity to eat more of the food and in exercise it is the capacity to exercise. When viewed as such it may not be surprising that with most currently available treatments the effect is diminished when the exposure is stopped. Intervening early with food when the allergy is not fully established may allow for more durable benefits and "eat away" at food allergy.

The ideal outcome of FA treatment is not clear or harmonized although efforts continue to standardize definitions and outcomes.^64^, ^65^ A recent review demonstrated that success varies based on the definitions used.^66^ Some trials define success as sustained unresponsiveness or remission.^67^ It is interesting that FA treatment is often held to a standard of maintained effect without the therapy, unlike other chronic disease therapies. For example, hypertension or diabetes medications are not expected to have a lasting effect when they are stopped. The concept of FA desensitization can be likened to exercise endurance where for most people the effect drifts down when the benefits diminish when exposure is stopped (Figure 5). If treatment is started early in the developement of allergy the effects may be more durable. Clinically, important goals of FA treatment can still be achieved even with this lack of permanence. The increased threshold provided by ongoing ingestion can cause a reduction in risk so that accidental exposures are unlikely to trigger severe, life‐threatening reactions. In the counseling of patients and families who may be curious about OIT, understanding patient goals and expectations is essential.^68^

While remission is a very high bar to ask of a treatment, it is valuable to know if a patient can safely miss doses or safely incorporate the food into their diet *ad lib*, as well as to drive the field towards the best achievable treatments. Currently, there are limited studies outlining predictors, both clinical and biomarker evidence, of treatment response or long‐term remission.^69^

QOL before, during, and after OIT in children has been examined. OIT can lead to significant logistical and psychosocial challenges. Patients and families must adhere to strict daily dosing schedules while managing ongoing anxiety about potential reactions. They may also need to find ways to adjust for variables such as travel, illness, or exercise. These are all factors that can place a considerable burden on both patients and caregivers during OIT. One large study looked at a group of children with allergies to a wide array of foods such as peanut, cow's milk, egg, tree nuts, and sesame and the FA QOL Questionnaire was used to assess QOL measures (emotional impact, food anxiety, social, and dietary limitation). Interestingly, they found that QOL scores typically improved on initiation of OIT to its maintenance. Some individuals did experience a decrease in QOL scores at the mid up‐dosing time but this reversed and improved as OIT continued. The benefits of OIT to QOL even extended to the smaller group of patients that reached a partial maintenance dose of allergic food. They identified that factors associated with an improved quality of life score included previous anaphylactic reactions before OIT, having a single FA, and a younger age when OIT was conducted.^70^

Overall, food immunotherapy is beneficial in increasing the reaction threshold for most food allergic individuals.^71^ The success of OIT is also very closely tied to patient compliance and adverse events resulting from the therapy. As there are safety concerns associated with the use of immunotherapy, detailed patient advice is needed.^68^ Unknowns and some future research directives for food‐based prevention of FA and therapies are summarized in Table 1. While dose has been considered above, another approach which may improve safety may be to change the route of delivery of foods.

**TABLE 1:** Important unknowns for food‐based prevention and treatment strategies.

Prevention of food allergy	Food based immunotherapy
Minimum sufficient dosing and frequency	Protocol considerations for optimal OIT: dosing, duration, frequency, when to de‐prescribe epinephrine,
Further information about other foods especially whether certain foods (? Cow's milk) may need an earlier introduction than 4–6 months of age	Role of adjuvants such as omalizumab (balancing cost)
Whether non‐IgE mediated allergy is increasing with early introduction	Clinical (age and other factors) and biomarker predictors of long‐term remission
	Long‐term risks of sustained therapy including (EoE)

#### 2.2.2 Sublingual and Epicutaneous Immunotherapy

Outside of OIT, there are new approaches in development for FA treatment.^71^, ^72^ Many studies are exploring other modalities of allergen exposure such as sublingual immunotherapy (SLIT) and epicutaneous immunotherapy (EPIT).

SLIT has shown promise in children and adults for FA desensitization.^71^, ^73^ One study examined children ages 1–4 years of age and found that peanut SLIT resulted in desensitization for most children after 36 months of treatment. It additionally found improvement in remission rates at 3 months post treatment compared to the placebo group.^73^ When compared to peanut OIT in the IMPACT trial, desensitization with SLIT occurred in a smaller proportion of participants (60% desensitization with SLIT and 71% with OIT). However, the side effect profile as well as the ease of administration are thought to be improved with SLIT compared to the typical doses and protocols used for OIT.

EPIT is under pharmaceutical development. A patch coated with peanut worn on the back underwent a phase 3 trial in children aged 4–11 years and found a significantly higher response rate in terms of peanut safely ingested compared to placebo after 12 months of treatment, but it did not meet a component of their initially defined primary outcome.^74^ A similarly designed study investigated the use of EPIT in children between the ages of 1 and 4 years and found that EPIT was superior to placebo.^75^ Additionally, the safety profile of EPIT was favorable with limited local application‐site reactions and high adherence.^75^

SLIT and EPIT have typically demonstrated a lower incidence of systemic reactions than OIT.^71^, ^76^ Most studies compare side effects reported from different independent trials. However, a head‐to‐head trial of SLIT compared to OIT demonstrated similar rates of reaction (29% in SLIT patients and 23% in OIT patients). It is important to recognize that reactions with OIT were more likely to be multisystem reactions. Another group examined the efficacy and safety profile of EPIT and SLIT to OIT. They found that although EPIT and SLIT demonstrated improved side effect profiles, they were less effective than OIT at achieving desensitization.^77^ These comparisons may also be limited by different doses used in these treatments, different populations, and different outcomes measured.

#### 2.2.3 The addition of biologics to food based treatments

Access to allergy and immunology care in Canada remains a challenge in many areas. According to the Canadian Medical Association, there were 219 practicing allergists in the country as of December 2019. Though this number has increased in the last 5 years, there are still a limited number of specialists, particularly outside of city centres.^78^ It is therefore imperative to keep striving for the safest and easiest treatments.

A reduction of IgE may allow for food‐based treatments for IgE‐mediated allergy to be easier and safer. The OutMATCH trial was a phase 3 trial that evaluated the efficacy and safety profile of 16 weeks of omalizumab as a monotherapy for FA or as a combination to OIT. Participants had one or more food allergies and were at least 1 year of age. The reaction threshold was increased for peanut, egg, milk and cashew compared to the placebo control group. This study also found that 80% of study participants could consume 1044 mg of one food, 69% of participants could consume 1044 mg of two foods, and 47% of participants could consume 1044 mg of three previously allergic foods. These findings have strong implications for individuals with multiple food allergies as taking one therapy (omalizumab) could provide protection and confidence towards eating foods containing numerous food allergens.^80^ In Canada, an ongoing RCT is assessing omalizumab as an adjunct to three food multi‐OIT. Children and young adults are randomized to 20 weeks of placebo injections, 8 mg/kg omalizumab monthly, or 16 mg/kg omalizumab monthly with an outcome of time to reach 500 mg food protein of each of 3 foods.^79^

### 2.3 Prevention and treatment as compared to the natural history of FA

While a detailed immunological review of FA is beyond the scope of this article, to contextualize prevention and treatment it is important to consider the natural history of FA. As an example, in the Health Nuts study, 1/3 of OFC proven peanut allergic infants were not allergic at 10 years of age. This finding suggests some of the resolution of peanut allergy seen when treating early may happen regardless of treatment. The natural resolution of peanut allergy was associated with decreasing sIgE and sIgG_4_ to peanut and Ara h 2. Interestingly, biomarker levels at diagnosis were not strongly associated with the natural history of peanut allergy,^81^ suggesting that maturation of allergy comes later than infancy for many. Early ingestion in infancy when not allergic^19^ and early treatment may prevent this allergy maturation^82^ and “eat away” at FA. A key difference between avoidance versus active ingestion is the high sIgG4 seen in active ingestion. Interestingly when the LEAP participants (non‐allergic infants) who were randomized to ingest peanut were followed for years, this sIgG4 began to decrease, even when still eating the food.^19^ In contrast, in OIT the IgG4 becomes elevated and remains elevated while sIgE drops over time showing that the immunological result of treatment differs from that of natural tolerance.^83^ Very long‐term OIT studies may shed further light on these laboratory trajectories.

## 3 CONCLUSION

Despite the rapid increase in the prevalence of food allergies, there have been many major advances in their understanding and management. There is greater recognition of how much food allergies impair the quality of life of children as well as their families, emphasizing the need for ongoing research and improvement in care, as there is still much that is unknown.

Advances in the use of food for prevention and treatment with immunotherapy as well as biologics for the treatment of FA have demonstrated promising outcomes, offering new pathways for improving tolerance and reducing allergic reactions. The continued development and refinement of prevention and therapies will be highly important for the future of FA care.

## AUTHOR CONTRIBUTIONS

**Yue Jennifer Du:** Writing – original draft; visualization; writing – review and editing. **Jessica C. Guo:** Writing – original draft; writing – review and editing. **Julia E. M. Upton:** Conceptualization; writing – review and editing; supervision; visualization; funding acquisition.

## FUNDING INFORMATION

Funding to summer students YJD and JCG was SickKids Food Allergy and Anaphylaxis Program.

## CONFLICT OF INTEREST STATEMENT

JEMU reports: Grants/Research Support: DBV Technologies, ALK‐Abello, Sanofi/Regeneron, Novartis. Consulting: Viatris, ALK‐Abello, Bausch Health, Pharming, DBV Technologies. Other: Employee of The Hospital for Sick Children, editorial board Allergy, Annals of Allergy, Asthma and Immunology, Journal of Food Allergy, associate editor AACI. The other authors have nothing to disclose.
